# Transverse Myelitis in a 72-Year-Old Male Presenting With Upper Extremity Weakness

**DOI:** 10.7759/cureus.65762

**Published:** 2024-07-30

**Authors:** Hannah Cox, Richard Virgilio, Luke Yuhico

**Affiliations:** 1 Internal Medicine, Edward Via College of Osteopathic Medicine, Auburn, USA; 2 Clinical Affairs, Edward Via College of Osteopathic Medicine, Auburn, USA; 3 Pulmonary and Critical Care, Hospital Corporation of America (HCA) Florida Fort Walton-Destin Hospital, Fort Walton Beach, USA

**Keywords:** plasmapheresis treatment, syndrome of inappropriate secretion of antidiuretic hormone (siadh), acute cervical transverse myelitis, longitudinally extensive transverse myelitis (letm), upper extremity weakness, neuromyelitis optica spectrum disorder, sjogren's, acute transverse myelitis (atm)

## Abstract

Acute transverse myelitis (TM) is a rare, acquired neuro-immune spinal cord disorder that can be idiopathic or related to a secondary disease. Clinical signs and symptoms include motor weakness, sensory alterations, and bowel or bladder dysfunction. Often TM occurs in the younger population or middle-aged adults. This patient’s presentation is unique in the fact that he does not fall into either of these age categories. In this case, a 72-year-old male with a past medical history of hypertension and type 2 diabetes mellitus presented to the emergency department due to a five-day history of worsening weakness of the upper extremities bilaterally. In addition, the patient reported a new onset of abdominal wall numbness. The patient reported being at a theme park a few days prior, denying any injuries and only complaining of neck discomfort during the car ride home. Labs and imaging were quickly ordered for diagnostic purposes. The patient was diagnosed with TM using magnetic resonance imaging (MRI), lumbar puncture, and clinical signs. The etiology was later discovered to be due to a new diagnosis of Sjögren's autoimmune disease. The patient was treated with high-dose intravenous steroids for five days while being monitored for any neurologic changes. The plan was to continue steroids by mouth once discharged from the hospital. Due to poor adherence to discharge instructions, the patient was readmitted after presenting to the emergency department with worsening symptoms. Physicians need to recognize and diagnose TM quickly, as some etiologies are treatable and can prevent further damage to the spinal cord.

## Introduction

The word myelitis refers to an inflammatory disease of the spinal cord [1]. The term transverse has been referred to the clinical symptom of a band-like horizontal area of sensory dysfunction and, more recently, in literature, to the position or width of inflammation within the spinal cord [2]. The disorder transverse myelitis (TM) particularly describes the characteristic findings of motor muscle weakness or paresis, sensory deficits, and autonomic dysfunction below the level of the lesion. Autonomic symptoms can include bladder and bowel incontinence, bowel constipation, sexual dysfunction, temperature dysregulation, or episodes of hypertension [2]. It can present in an acute or subacute manner, leaving patients with moderate to severe disabilities in up to two-thirds of the cases [1-3]. A typical coarse of TM develops over hours to days and can worsen over days to weeks [2]. In 60% of cases, idiopathic TM affects the cervical region, with the thoracic region being affected in 33% of cases [4]. 

TM is categorized as an inflammatory demyelinating disorder. These types of diseases are differentiated by their distribution of inflammation as well as the rates of recurrence [3]. An article published by Bhat et al. found that the incidence of TM is between 1.34 and 4.6 per million per year, with a bimodal peak of cases between the ages of 10-19 and 30-39 [5]. With previously low incident rates, it can be difficult for emergency departments to diagnose and recognize TM with patients presenting minimal signs [6]. A study by Huh et al. was conducted to help improve the ability to identify TM in the emergency department. According to this study, sensory changes were identified in 45 out of 46 patients (97.8%), motor weakness in 33 patients (71.7%), and autonomic dysfunction in 35 patients (76.1%) [6]. Recognizing these symptoms right away can help diagnose and treat TM appropriately to decrease further damage. 

It is also important to distinguish between TM and compression of the spinal cord. Both can present with similar symptoms but are treated drastically differently, with compression requiring emergent surgery [2]. After a compressive myelopathy has been ruled out, the etiology of TM must be investigated if it can be identified [2]. Non-compressive myelopathies can be categorized into ischemic, paraneoplastic, infectious, acquired demyelinating autoimmune disorders, drug- or toxin-induced, or systemic autoimmune diseases [1,3]. The most common autoimmune diseases associated with TM include systemic lupus erythematosus (SLE), Sjögren's syndrome (SS), sarcoidosis, Behçet's disease, antiphospholipid syndrome (APS), as well as other connective tissue diseases. TM may be the first manifestation of these diseases, as well as the initial symptoms of multiple sclerosis (MS) and neuromyelitis optica (NMO). Unfortunately, in some cases, the etiology of TM cannot be determined and is labeled as idiopathic TM [3]. This diagnosis of idiopathic TM occurs in about 15% to 30% of patients [1].

A common presentation of TM is the combination of TM and optic neuritis. This is termed neuromyelitis optica (NMO) [3]. The anti-aquaporin-4 (anti-AQP4) antibody has been identified in patients with NMO but also in patients with idiopathic recurrent acute transverse myelitis (ATM) and longitudinally extensive transverse myelitis (LETM) [7]. LETM refers to inflammatory lesions on MRI that extend beyond three or more vertebral segments. In this study, researchers wanted to describe the frequency of this antibody, as well as its influence on long-term prognosis. They found that the frequency of anti-AQP4 in recurrent ATM was 26.9%, with results increasing to 41.2% among those with LETM. They also found no significant influences on morbidity with this antibody [7].

Management of ATM initially begins with intravenous anti-inflammatory drugs to prevent further damage and decrease swelling. Immunosuppressive drugs, such as cyclophosphamide, azathioprine, and intravenous immune globulin, may also be used to treat TM. Plasma exchange (PLEX) is an alternative treatment for TM in patients who do not respond well to IV corticosteroids. Finally, to decrease the reoccurrence of TM in patients with NMO, a monoclonal antibody, such as rituximab, has been shown to be effective [4]. Rituximab may be suitable for those patients with concurrent NMO or MS. Long-term prognosis depends heavily on the etiology of TM. Based on the underlying etiology, some patients have no lasting effects, while others are disabled to varying degrees [2].

## Case presentation

A 72-year-old Hispanic male with a past medical history of chronic hypertension and type 2 diabetes mellitus presented to the emergency department due to a five-day history of worsening weakness of the proximal and distal upper extremities bilaterally. The patient reports having been at a theme park a few days prior. He denies any injuries while at the theme park but does report noticing neck discomfort while traveling home from the theme park. The patient denies any recent illness or exposure to sick contacts. He reported originally presenting to a freestanding clinic, but telehealth neurology recommended transfer to a higher level of care. At the freestanding clinic, the patient was started on acetaminophen with codeine for neck pain and methylprednisolone due to his symptoms presenting as an inflammatory condition. While in the emergency department, he reported a new onset of upper abdominal wall numbness, with no difficulty in breathing. The patient denied tobacco smoking and illicit drug use but reported the consumption of approximately one beer per week. He denied any known drug allergies or significant past family history. His past surgeries include a varicocelectomy. His current medications included lisinopril, propranolol and metformin, for hypertension and type 2 diabetes mellitus, respectively. Upon further questioning, he reported bilateral upper extremity weakness, abdominal wall numbness, and constipation. He reported the constipation had been ongoing for the last three days. He denied any fever, headache, vision change, facial weakness, slurred speech, chest pain, or lower extremity weakness or numbness.

The pertinent findings from his physical exam included decreased musculoskeletal strength in his upper extremities, decreased pin-prick sensation mostly on the upper abdominal wall, and decreased sensation to temperature in the right arm. The patient’s upper extremity strength presented more distal than proximal and was rated a three out of five, and he was unable to perform fine motor tasks. The patient’s lower extremities were not involved. The patient was alert and oriented to person, place, time, and event with normal speech, and cranial nerves II through XII were intact.

Following the protocol for a patient presenting with neurologic symptoms, he was sent for imaging and laboratory orders, and the neurology team was consulted immediately. The patient’s pertinent abnormal values and all other values were within the normal range (Table 1). 

**Table 1:** Lab values. BUN: blood urea nitrogen.

Lab test	Patient’s value	Reference range
WBC	14.1 K/mm^3^	4.23-9.07 K/mm^3^
RBC	4.59 M/mm^3^	4.63-6.08 M/mm^3^
Hgb	13.4 gm/dl	13.7-17.5 gm/dl
Neutrophil	12.00 K/mm^3^	1.78-5.38 K/mm^3^
Neutrophil %	85.2%	34.0-67.9
Lymphocyte %	11.2%	21.8-53.1%
BUN	23 mg/dL	7-18 mg/dL
Alkaline phosphatase	43 Units/L	46-116 Units/L
Glucose	119 mg/dL	74-106 mg/dL
Hgb A1C	5.9%	0-5.71%

**Figure 1:**
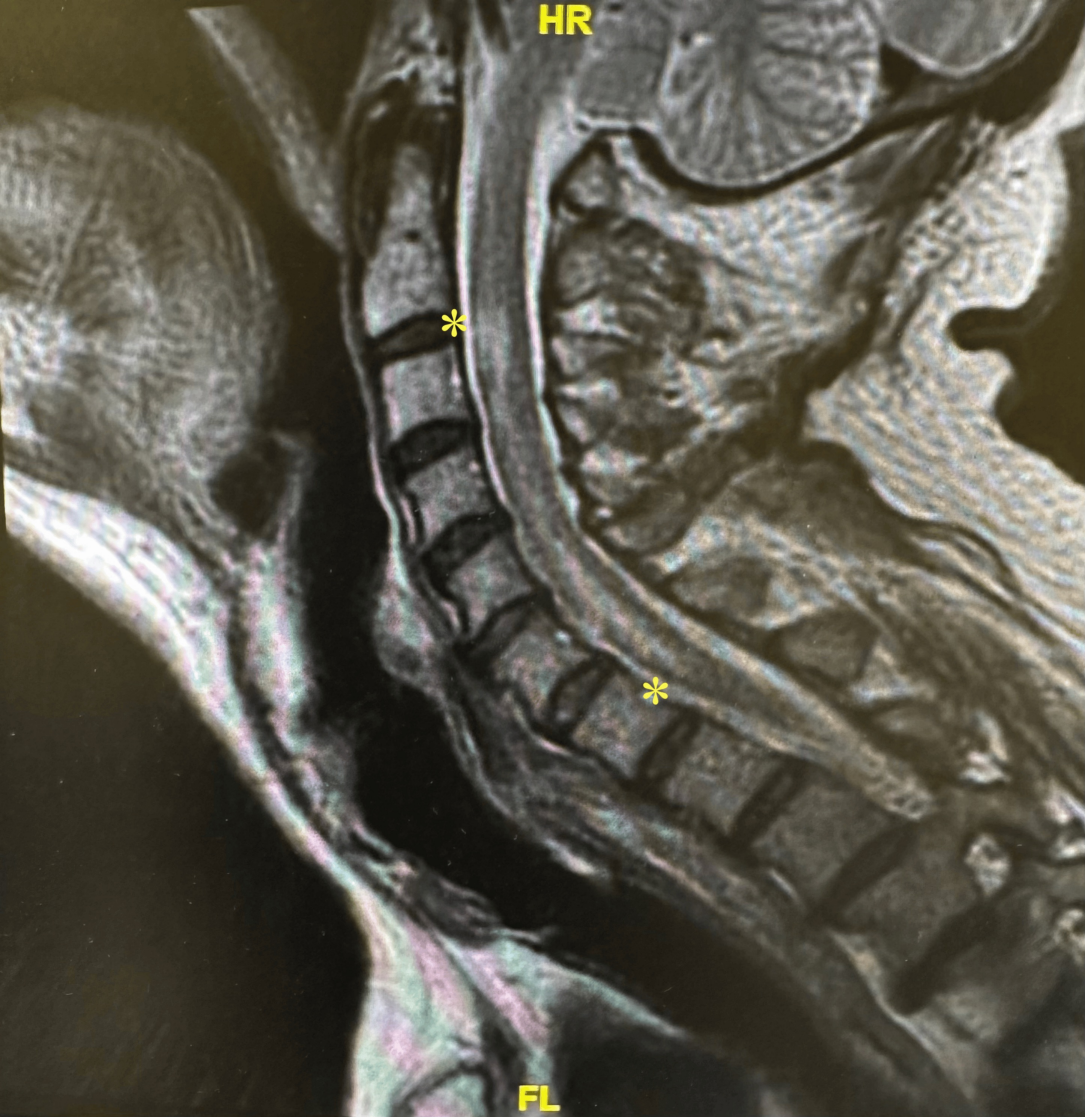
MRI with asterisk showing a diffuse T2 from cervical spine two through seven. MRI: magnetic resonance imaging.

Chest x-ray, head and neck computed tomography angiography (CTA) with and without contrast, brain computed tomography (CT) without contrast, and brain magnetic resonance imaging (MRI) were ordered, and all revealed normal results. On admission day two, a cervical spine MRI was ordered and revealed a diffuse T2 signal in the anterior third of the cord from cervical spine 2 through 7 (Figure 1).

After the results of the MRI, on admission day three, the patient was diagnosed with transverse myelitis. The patient was admitted to the medical telemetry unit and had frequent neurological evaluations as per neurological team advice. On admission day one, the patient was screened for Lyme disease, systemic lupus erythematosus, Sjögren's, vitamin B12 deficiency, and a copper deficiency with negative results except for positive SS-A/Ro (>8.0 out of 0-0.9) and antinuclear (ANA) antibodies, suggesting an autoimmune-related cause. Due to the longitudinally extensive nature of the lesion, neuromyelitis optica must be entertained. The patient was tested for the NMO-IgG antibodies and AQP4 antibodies; although the results took some time due to them being send-out labs, ultimately both resulted negative. On admission day three, treatment began with intravenous methylprednisolone for five days. His white blood cell count was measured at 11.96 m/mm^3^ (reference: 4.23-9.07 m/mm^3^) and red blood cell count at 4.55 m/mm^3^ (reference: 4.63-6.08 m/mm^3^) consistent with leukocytosis and anemia, respectively. His symptoms began to improve, and laboratory results became normalized on admission day five. The etiology of TM was attributed to Sjögren's due to the patient’s positive SS-A/Ro laboratory result. The patient was discharged on admission day five and prescribed methylprednisolone to be taken at home.

Four days after being discharged, the patient represented to the emergency department with worsening symptoms of weakness and paresthesia of the upper extremities and abdomen. The patient stated he was never discharged home with methylprednisolone, despite the discharge summary stating he was; therefore, he never began the medication. He also noted to have angina pectoris at this time. Cardiac investigations were normal, including troponin T, troponin I, as well as electrocardiogram. His blood pressure in the emergency department reached a peak of 213/91, and he received intravenous 10 mg of hydralazine for immediate blood pressure control. The patient did not report any associated headaches or visual symptoms. Due to this episode of hypertensive urgency, the patient was prescribed IV hydralazine 10 mg every four hours as needed to maintain a systolic blood pressure of less than 170. While in the emergency department, routine labs were ordered and the abnormal values are depicted in Table 2.

**Table 2:** Lab values.

Lab test	Patient’s value	Reference range
Sodium	124 mmol/L	136-145 mmol/L
Chloride	87 mmol/L	98-107 mmol/L
Creatinine	0.36 mmol/L	0.6-1.0 mmol/L
Alkaline phosphatase	50 U/L	53-128 U/L
Total protein	6.1 g/dL	6.4-8.1 g/dL
WBC	12.6 k/mm^3^	3.1-8.8 k/mm^3^
Neutrophil	10.40 k/mm^3^	1.78-5.38 k/mm^3^
Neutrophil %	82.2%	34.0-67.9%
Lymphocyte %	12.0%	21.8-53.1%
Glucose	131 mg/dL	74-106 mg/dL

Because of the patient’s very low sodium level, the nephrology team was consulted at this time, and he was transferred to the intensive care unit (ICU) for treatment of hyponatremia. A lumbar puncture was performed on admission day two, which revealed cerebrospinal fluid protein and segmented neutrophils elevated, with all other values within normal limits. He was managed initially on 3% intravenous saline for one day due to the rate of which sodium was decreasing. Sodium levels were monitored every two hours by the nursing staff. The patient's sodium continued to trend down at 122 mmol/L, 122 mmol/L, 118 mmol/L, 128 mmol/L, 118 mmol/L, 118 mmol/L, 117 mmol/L, and his serum osmolarity measured at 255 msom/kg (reference: 275-295 msom/kg), urine sodium <10 mmol/L, and urine osmolality at 791 mosm/kg (reference: 300-900 msom/kg) and was diagnosed with the syndrome of inappropriate of antidiuretic hormone (SIADH) on admission day four. Also, on admission day four, the patient became altered, and an arterial blood glass showed that the patient was severely hypercapnic and acidotic, with a pCO_2_ >121 mmHg (reference: 35-45 mmHg) and pH of 6.96 (reference: 7.35-7.45). At this time, the patient was placed on BiPAP for management. His hyponatremia was resolved on admission day six after treatment with fluid restriction per the nephrology team. To rule out infectious causes of TM, the patient was also managed on intravenous vancomycin at 1,000 mg every twelve hours, which was increased to 1250 mg for the second and third doses, for a total of three doses. Due to a lack of improvement, he was further given intravenous cefepime 1 g every twelve hours for five days for the newly diagnosed TM.

The patient responded poorly to high-dose intravenous steroids, and plasmapheresis was started on admission day five for additional treatment. He was treated with five courses of plasmapheresis on admission days five, six, nine, eleven, and thirteen. These treatments can take up to two to three hours at a time. During this time, there were no adverse reactions. Throughout this patient's stay, he was monitored and treated daily. No major events occurred during this time. He gradually improved and was discharged on admission day thirty-seven. The plan of care was to be discharged to a long-term acute care facility as well as to continue oral steroids and taper for an additional three months.

## Discussion

Although this patient presented with mild symptoms and did not fall into the traditional age category for the presentation of TM, this case of TM adds to today’s literature of TM due to autoimmune-related etiologies. While being worked up for an etiology in the hospital, this patient tested positive for the SS-A/Ro antibody, consistent with Sjögren's syndrome. This was believed to be the cause of his TM. TM can be the first presentation of these autoimmune diseases. Sjögren's syndrome is an autoimmune connective tissue disease that primarily affects the exocrine glands but also has extra-glandular manifestations [8]. In this disease, the exocrine glands are destroyed, resulting in symptoms of dry eyes (also referred to as keratoconjunctivitis) and dry mouth (xerostomia). The patient, in this case, was questioned about the symptoms, with only the patient's daughter recalling minimal symptoms in the past. The most common extra-glandular involvement is the nervous system. Sensory peripheral neuropathy is the most common neurological complication, with the central nervous system being much less common (2-25%). Among spinal cord involvement in SS patients, the most common form is ATM, with the cervical region being the primary location [8]. This is consistent with the patient in this case, which further solidifies the findings from this patient's workup.

Due to this patient’s lesion on MRI extending from C2-C7, it is appropriate to work this patient up for the NMO-IgG antibodies as well as anti-AQP4. LETM is defined as inflammation in the spinal cord causing T2 hyperintensity on MRI extending across three or more vertebrae. These two specific antibodies are strongly associated with LETM, although obtaining these laboratory results can be difficult due to cost or not being available locally [9]. The test for these specific antibodies was not available in the patient’s community hospital; therefore, they had to be sent out. Fortunately, the results were available when the patient presented a second time to the emergency room. Aquaporin-4 is the main channel in the astrocytes of the central nervous system for water transport. The pathogenesis of NMO and these antibodies involves autoantibodies binding to aquaporin-4, disrupting its normal function, causing the blood-brain barrier to become hyperpermeable, damaged, and eventually leading to spinal cord and optic nerve demyelination [10].

While continuing to monitor this patient, an abnormal sodium laboratory result raised new questions and interest. Due to sodium levels continuing to trend down despite replacement, the patient was diagnosed with SIADH. SIADH is a potential cause of hyponatremia of the central nervous system, but being associated with NMO is rare. Although this patient was not diagnosed with NMO, one study states that patients can show seronegative aquaporin-4 antibody results [11]. With continued treatment of 3% saline infusions and close monitoring, the patient’s hyponatremia resolved.

This patient was managed on intravenous steroids and plasmapheresis while in the hospital. Due to failed treatment with corticosteroids alone, an additional treatment regimen had to be added. Plasma exchange (PLEX), also known as plasmapheresis, is a treatment in which the plasma is separated from inflammatory factors, complement factors, and cytokines. PLEX is effective in about 45% of steroid-refractory cases [12]. The goal of PLEX is to remove any humoral factors [2] and autoreactive antibodies [4] over a ten-day period. To receive the best results from PLEX, it is best to initiate treatment within two weeks of symptoms. The standard recommendation for treating a patient with TM is the administration of high-dose intravenous corticosteroids for three to seven days [2]. Following proper treatment, the patient continued to improve enough to be discharged home, while continuing corticosteroids orally.

## Conclusions

Due to the various presentations and etiologies of TM, it is important to recognize and treat it in a timely manner. A patient presenting with motor, sensory, or autonomic symptoms should be investigated for TM. Some patients may even present with subtle symptoms of TM. The various etiologies of TM should be investigated to adequately treat the patient. Physicians should be aware of this disease presentation due to the poor prognosis if not identified and treated properly.
